# The Antibiotic Guardian campaign: a qualitative evaluation of an online pledge-based system focused on making better use of antibiotics

**DOI:** 10.1186/s12889-017-4552-9

**Published:** 2017-07-11

**Authors:** Joanna May Kesten, Alex Bhattacharya, Diane Ashiru-Oredope, Maya Gobin, Suzanne Audrey

**Affiliations:** 1 The National Institute for Health Research Health Protection Research Unit in Evaluation of Interventions, School of Social and Community Medicine, University of Bristol, Bristol, UK; 2 The National Institute for Health Research Collaboration for Leadership in Applied Health Research and Care West (NIHR CLAHRC West) at University Hospitals Bristol NHS Foundation Trust, Bristol, UK; 3 Antimicrobial Resistance Programme, Public Health England, London, UK; 4 Field Epidemiology Service, Public Health England, Bristol, UK; 5 School of Social and Community Medicine, University of Bristol, Bristol, UK

**Keywords:** Antimicrobial resistance, Antibiotic, Pledge system, Qualitative research, Evaluation, Behaviour change, Public health

## Abstract

**Background:**

The Antibiotic Guardian Campaign was developed to increase commitment to reducing Antimicrobial Resistance (AMR), change behaviour and increase knowledge through an online pledge system for healthcare professionals and members of the public to become Antibiotic Guardians (AG). This qualitative evaluation aimed to understand AG experiences of the campaign and perceived impact on behaviour.

**Methods:**

Ninety-four AGs (48 via a survey and 46 who had agreed to future contact) were invited to participate in a telephone semi-structured interview. The sample was based on self-identification as a healthcare professional or a member of the public, pledge group (e.g. adults, primary care prescribers etc.), pledge and gender. Interviews explored how participants became aware of the campaign, reasons for joining, pledge choices, responses to joining and views about the campaign’s implementation. Interviews were analysed using the Framework Method.

**Results:**

Twenty-two AGs (10 healthcare professionals and 12 members of the public) were interviewed. AGs became aware of the campaign through professional networks and social media, and were motivated to join by personal and professional concern for AMR. Choice of pledge group and pledge were attributed to relevance and potential impact on AMR and the behaviour of others through pledge enactment and promotion of the campaign. Most AGs could not recall their pledge unprompted. Most felt they fulfilled their pledge, although this reflected either behaviour change or the pledge reinforcing pre-existing behaviour. The campaign triggered AGs to reflect on AMR related behaviour and reinforced pre-existing beliefs. Several AGs promoted the campaign to others. Responding collectively as part of the campaign was thought to have a greater impact than individual action. However, limited campaign visibility was observed and the campaign was perceived to have restricted ability to reach those unaware of AMR.

**Conclusions:**

AGs were motivated to reduce AMR and most felt they fulfilled their pledges although for many this appeared to be through reinforcement of existing behaviours. We recommend that the campaign engages those without pre-existing knowledge of AMR by increasing its visibility, capitalising on the diffusion of its message and including more awareness-raising content for those with limited AMR knowledge.

**Electronic supplementary material:**

The online version of this article (doi:10.1186/s12889-017-4552-9) contains supplementary material, which is available to authorized users.

## Background

Antimicrobial resistance (AMR), the process of bacteria and other pathogens evolving to become resistant to drugs and medicines, is a significant public health concern [1] worsened by the inappropriate use of antibiotics [1]. The use of antimicrobials is informed by a number of complex interacting factors including awareness of appropriate antimicrobial use, expectations of antimicrobial prescription and communication between healthcare professionals and patients [2]. In the 2013 Eurobarometer report on AMR, 52% of those surveyed in the UK correctly replied that antibiotics do not kill viruses, however colds and flu were amongst the five leading reasons for taking antibiotics [3] suggesting there is a mismatch between knowledge and behaviour. Increasing understanding of the consequences of inappropriate antibiotic use has been highlighted as important for creating a social norm for antibiotics to be seen as a “last resort” p6 [4].

It is clear that multi-level action, targeting healthcare providers and members of the public [2], is needed to “prevent overuse, misuse and abuse” (p76) of antimicrobials [5]. “Responsible use” policy interventions to address AMR attempt to “ensure that patients receive the right treatment at the right time, use these drugs appropriately and benefit from them” (p285) [6]. Public awareness campaigns are one type of responsible use intervention to improve the public’s knowledge of suitable antibiotic use and causes of AMR. Educational interventions targeting the public and healthcare professionals are recommended in the World Health Organisation (WHO) strategy for containing AMR [2]. It is difficult to assess the effects of public health campaigns on AMR, antibiotic consumption and prescribing, however a review of campaigns suggests they can contribute to improved antibiotic use [7].

The European Antibiotic Awareness Day (EAAD), which takes place annually on 18th November, aims to increase awareness about the threat of AMR to public health and encourage appropriate antimicrobial use [8]. The EAAD is supported by a website, social media and various educational materials [8] and has received widespread support from participating countries [6].

As part of the 2014 EAAD, an online pledge system called the Antibiotic Guardian (AG) Campaign, was launched in September in the United Kingdom (UK) to increase people’s commitment to reduce AMR [9]. Public Health England (PHE) and the British Society for Antimicrobial Chemotherapy developed the initial concept for the AG website design and logo and provided funding for the website development. The development of the campaign was led by PHE in collaboration with a multidisciplinary committee of animal and human health professionals and representation from members of the public across the UK from 26 organisations. The campaign was publicised by the PHE AMR and communications team, through free social media and press release promotions, and promoted by a volunteer network of organisations including representatives from the NHS (primary and secondary care), directors of public health, local authority health and wellbeing boards and professional organisations.

**Fig. 1:**
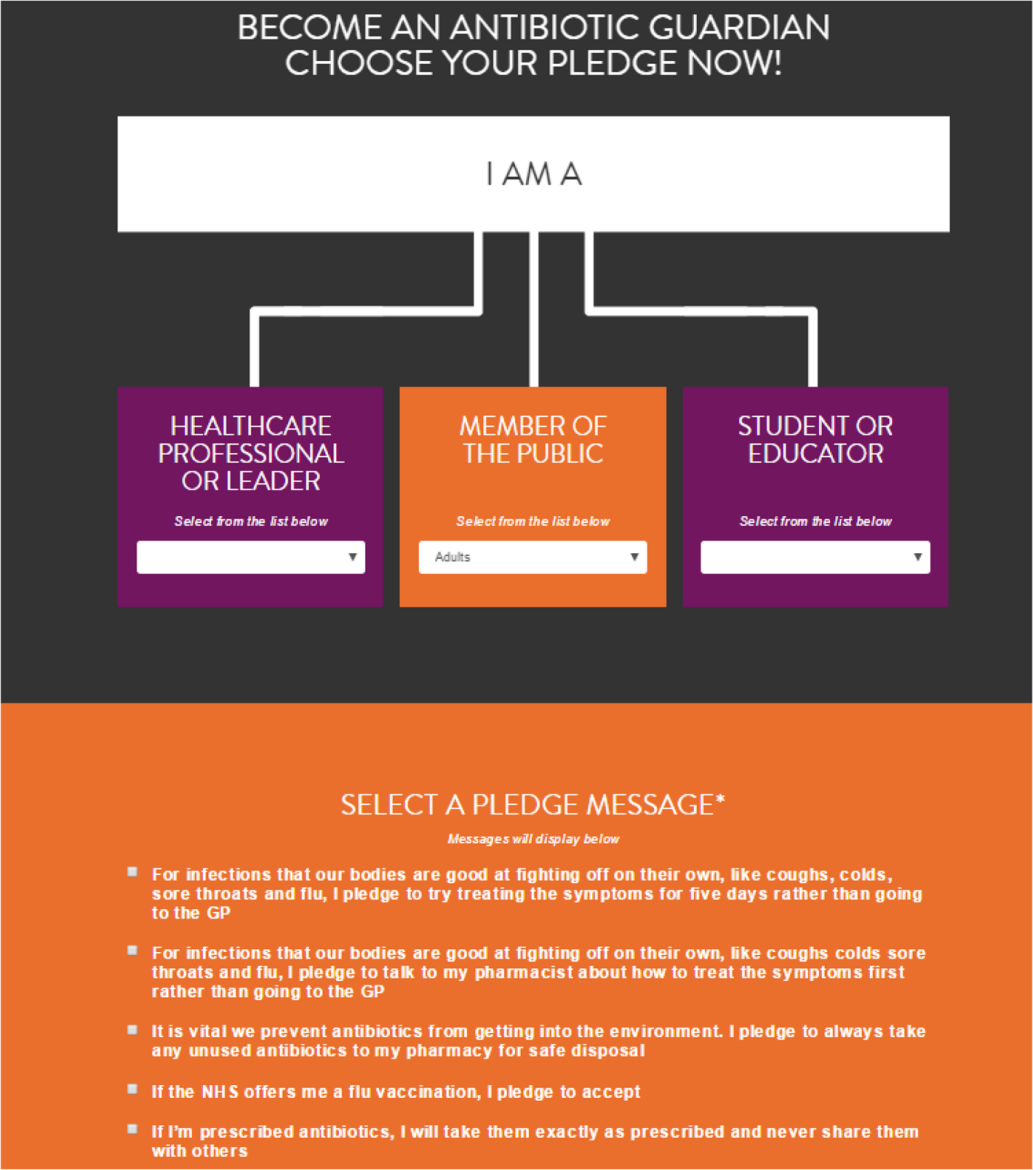
Antibiotic Guardian Campaign website: pledge group and pledges (http://antibioticguardian.com/)

A detailed description of the campaign has been published previously [10, 11]. Briefly, AGs choose from a number of pledge groups as either a ‘Healthcare professional or leader’ (e.g. Primary Care Prescribers, Nurses, Dentists etc.) or ‘Member of the public’ (e.g. Adults, Families and Pet Owners etc.) (Fig. 1). Within the pledge groups AGs select one pledge from lists offering between three and eight options (Additional file 1). The campaign website includes a video, aimed at the public, and written resources explaining the AMR issue. The initial target of 10,000 pledges by 30th November 2014 was achieved and in April 2017 the total number of pledges was ~46,000.

The campaign extends the EAAD goal of raising awareness and seeks to change behaviour through the use of pledges and support for collective action or responsibility (identifying as an AG, visiting the website and being part of a group of AGs) by addressing the intention-behaviour gap in relation to the appropriate use of antibiotics [12]. The intention-behaviour gap describes a situation in which an individual is committed to a goal but does not achieve it. It has been suggested that this gap can be addressed by supporting people to translate goal intentions into behavioural action in an “if-then” format [13]. For example, one of the campaign’s pledges is “For infections that our bodies are good at fighting off on their own, like coughs, colds, sore throats and flu, I pledge to try treating the symptoms for five days rather than going to the GP” (General Practitioner). Setting implementation intentions increase the likelihood of goal attainment by supporting the automatic trigger of certain actions in pre-determined situations [14]. Meta-analyses of experimental studies have shown medium to large positive effects (d = 0.65) of implementation intentions on goal attainment [15] and medium to large changes (d=0.66) in behavioural intentions has shown small to medium changes (d=0.36) in behaviour [16]. Pledges have been recommended in a literature review and behavioural analysis of behaviour change and antibiotic prescribing [4] and have been employed in a number of other situations to support behaviour change [17–20]. The evaluation of previous public campaigns addressing appropriate antibiotic use have either been absent or variable in quality [7].

The NIHR Health Protection Research Unit in Evaluation of Interventions was commissioned by PHE to conduct a mixed methods evaluation of the AG campaign to assess whether its goals were achieved. The quantitative component of this evaluation [10] analysed 2478 AG responses to an online questionnaire, completed 5 months after the launch of the campaign, assessing self-reported knowledge and behaviour after joining the campaign. This evaluation found self-reported evidence for an increase in AG knowledge about and commitment to addressing AMR, behaviour change in line with pledges after joining the campaign and willingness to promote the campaign to others [10]. PHE conducted a quantitative process evaluation to examine the reach of the campaign in its first 6 months (August 2014 to January 2015) and found that the majority of AGs identified as female (61%) and healthcare professionals (69%) [11].

The aim of this qualitative evaluation is to provide a more detailed understanding of participants’ views and experiences of the campaign and the perceived impact on behaviours related to AMR including health seeking behaviours and antibiotic consumption.

## Methods

### Sample and data collection

Semi-structured interviews were conducted to elicit understanding of AGs views and experiences of the campaign. Due to the geographical spread of AGs across the UK, one-to-one telephone interviews were the chosen method of data collection. The sample size was determined pragmatically with an initial target of approximately 30 AGs (~15 ‘members of the public’ and ~15 ‘healthcare professionals or leaders’). A third option, ‘Student or Educator’, was added later in the campaign: Students were previously included within both ‘Members of the public’ and ‘Healthcare professional or leader’, but the Educator group was new to the campaign and was not included in this evaluation. Within the pledge groups we sought to achieve a sample with a range of self-selected pledges and a gender balance similar to the main AG cohort. A purposive sampling strategy was designed to capture these characteristics to achieve a sample reflecting the AG cohort. In addition, a meta-analysis has found that women are more likely to receive an antibiotic prescription than men in primary care suggesting that there are gender differences in antibiotic prescribing behaviours which may translate into different interactions with the campaign [21].

AGs were invited in small batches via email and provided with participant information sheets between August 2015 and January 2016 (10–16 months after the campaign launch). If an invitation was declined, or no response received, another individual was invited with similar characteristics.

AGs were recruited for interview from two samples of the main AG cohort. Sample 1 consisted of 57 AGs who had agreed to receive an invitation to a qualitative study when they were sent the questionnaire as part of the quantitative evaluation (*n* = 9016, 0.63% response rate). Within this sample, 63% selected pledges as ‘healthcare professionals or leaders’ and 37% as ‘members of public’. Eight AGs were excluded as their postcode indicated they were non-UK residents, and one was excluded for not selecting a pledge. Of the 48 eligible individuals, 10 ‘members of the public’ and eight ‘healthcare professionals’ were interviewed (38% recruitment rate) (Additional file 2). To increase the sample size and representation of pledge groups, in Sample 2 we purposively sampled from those who had agreed to be contacted about their pledge in the future when they joined the campaign (*n* = 46) (Additional file 2). This yielded an additional four AGs (8.7% recruitment rate) representing pledge groups not recruited in Sample 1 (farmers, pet owners, primary care prescribers and students).

The interview topic guide explored how AGs became aware of the campaign, reasons for signing up, the choice of pledge group and pledge, and responses to becoming an AG including the extent to which the pledge was followed. In particular, the evaluation sought to understand if AGs changed their approach to managing illness (either personally or for family members or patients), their use or prescription of antibiotics and whether they promoted the campaign to others. Lastly, AGs were asked to reflect on the overall impact and value of the campaign in the UK and to feedback on the campaign’s content and design.

Verbal informed consent was audio recorded using an encrypted recording device from all participants before the interview. This approach minimised participant burden by avoiding the inconvenience of returning (by either post or email) written informed consent forms before the interview. Written and verbal consent are ethically comparable. The University of Bristol, Faculty of Health Sciences granted exemption from requiring ethical permission for this service evaluation.

### Analysis

Interviews were transcribed verbatim and anonymised. The Framework Method [22], a form of thematic analysis, was undertaken with the support of QSR NVivo Version 10. Analysis began with a process of ‘familiarisation’ with the audio recordings and transcripts. Initial codes, which summarise and interpret the data, were then systematically applied to the first few transcripts by the main coder (JK) and a second coder (SA). These initial codes were iteratively refined and combined to produce an agreed coding framework which was applied to all the remaining transcripts. As new or redundant information was identified during this process, revisions were made to the coding framework. The coded data was then inserted into a Framework matrix in NVivo which plots the codes against each participant. The volume of data was condensed and summarised by theme and subtheme for each transcript. These summaries captured the meanings in the data and included illustrative quotes. Summarising the data in the matrix facilitated interpretation of the data [22]. This method was selected to facilitate reflections on salient themes, and similar or divergent perspectives between participants [22].

## Results

Twenty-two AGs representing a diverse range of pledge groups including 12 ‘members of the public’ and 10 ‘healthcare professionals or leaders’ were interviewed (Table 1). This represents a recruitment rate of 23.4% (22 out of 94 invited). Secondary care prescribers and nurses were not represented in the sample. The mean interview length was 51.8 min (range 29 to 116 min). In the past year, antibiotics had been used personally by seven AGs and by a family member of six. Five AGs had prescribed or administered antibiotics (e.g. to livestock) in the past year (Table 1). Six healthcare professional AGs were unable to prescribe antibiotics in their role (2 AIPs, pharmacist, podiatrist, student, vet). The recruited sample had a higher proportion of female AGs (81%) and a lower proportion of HCPs (45.4%) compared to those invited (77.6% female, 70.2% HCP) and the total population of AGs described in the quantitative process evaluation (61% female, 69% HCP) [11].

**Table 1:** Interview participant characteristics

Antibiotic Guardian Campaign pledge group	N
Members of the public	12
Adult	8
Families	2
Farmer	1
Pet owner	1
Healthcare professional	10
Antimicrobial/Infection Prevention and Control Specialists (AIP)	2
Dentist	2
Student (medical)	1
Other healthcare provider (podiatrist)	1
Pharmacist	1
Primary care prescriber	1
Vet	2
Gender	
Male	4
Female	18
Use of antibiotics in the past year^a^	
Yes (personal)	5
Yes (family member or livestock)	4
Yes (prescribed antibiotics)	2
Yes (personal and family)	1
Yes (personal, family and prescribed)	1
Yes (prescribed and family)	1
No use or prescription of antibiotics	12
Total	22

^a^Six AGs pledged as healthcare professionals but were unable to prescribe antibiotics in their current role (2 AIPs, pharmacist, podiatrist, student, vet)

The four main themes and associated subthemes are presented in Table 2. Each theme is described below and supported by anonymised quotes with participant descriptors of pledge group, gender and interview number.

**Table 2:** Qualitative themes and subthemes

Themes	Subthemes
Campaign awareness	• Employer and professional networks • Invitation (e.g. via email) • Social media • Can’t remember • News bulletins • Radio
Campaign decision making	Reasons for signing up and initial impressions • Interest / concern about AMR • Lack of awareness / interest in raising awareness of AMR • Pre-existing knowledge of correct use of antibiotics • Moral duty and personal responsibility • Willingness and ability to help • Furthering career/professional interest • Likened to signing a petition • Blame felt regarding antibiotic resistance • Failure of current messages to change mind sets • Access and use campaign promotional materials • Previous illness experience • Minimal effort / easy to sign up Pledge group • Most pertinent / relevant • Qualifying for more than one group • Ability to have an impact • Other pledge group suggested Pledge choices • Pledge matched behaviour already performed • Relevance of pledge • Greatest perceived impact • More than one pledge relevant • Specific pledge feedback • Different or amended pledges preferred/suggested • Simplicity/clarity of information • Age of child • Breadth of pledge
Pledge recall	N/A
Impact of campaign	• Limited personal impact of campaign • Value of the campaign • Number of people signed up • Raised awareness • Reduced antibiotic use • Unintended consequences • Time needed for impact • Reduced antibiotic resistance • Reducing workload of GP • Reaching a wider audience • Visibility of campaign

### Campaign awareness

Most AGs struggled to remember where they had heard about the campaign and suggested a number of channels through which they may have become aware of it including professional networks (e.g. email invitations), and social media.

*I think it was probably emails from professional organisations, so the British Society of Antimicrobial Chemotherapy, PHE.* (Antimicrobial/Infection Prevention and Control Specialists (AIP) Female Interview 18)

### Campaign decision making

The campaign requires AGs to make a number of decisions; firstly, whether to join the campaign and subsequently which pledge group and pledge to select.

#### Reasons for signing up and initial impressions

The majority of AGs were motivated to join the campaign by their pre-existing interest and concern about AMR related issues.

*My reason comes from being a vet, in that I’m concerned that antimicrobials will be removed from the veterinary sector, and I believe it’s important that we need to use them properly, because animals need them as much as humans to avoid affecting animal welfare. So it came from that - is that I want to keep antimicrobial use with animals and not just being reserved for humans.* (Vet Female Interview 13)

*Well I thought it [the campaign] was about over prescribing and over use and I totally agree that something needs to be done because if you keep using antibiotics at the rate we are they will not do us any good.* (Adult Female Interview 4)

AGs agreed with the message behind the campaign and wanted to “play their part” and set a good example for others. The campaign was perceived as encouraging collective responsibility for addressing the AMR issue. For example, one AG likened it to the “Big Society” UK government initiative in which a larger proportion of responsibility for running society is devolved to local communities and volunteers. Responding collectively as part of the campaign was anticipated to have a greater impact than individual action.

*I’d love to do something about it [AMR], but it’s really hard when you’re just one person [laughs]. So signing up to campaigns like this sort of does make you think, right, okay, well it’s sort of demonstrating that the general public does actually care about these things.* (Families Female Interview 5)

*I think it’s because when I was looking at it and thinking that it was such a good idea that people were taking responsibility for their own actions and that we could all be part of it – I suppose it’s feeding into this kind of Big Society idea – I thought, if I just look at this and don’t actually do it, then that makes me worse than if I didn’t do it ‘cause I wasn’t aware of it. So I felt I needed to actually take an action from it because it is something which is so important and it is something that I agree with, so I thought (…) it’s almost like signing one of these petitions.* (Adult Female Interview 11)

As described in the extract above, the campaign was likened to signing a petition and the number of pledges was equated to the effectiveness and importance of both the campaign and the AMR issue. By joining the Campaign, AGs hoped the campaign would grow and increase its impact.

*Because a bit like signing a petition, (…) the bigger a group of people there are who are even silently campaigning about this, even if it’s the fact that they don’t use antibiotics themselves (…) it’s just getting the numbers up to show that this is such an important cause.* (Adult Female Interview 7)

*I guess the reality is the more people who sign up, the wider it will spread.* (Pet owner Female Interview 21)

There was a perception among AGs that the general public were not fully aware of the AMR issue, did not take the issue seriously enough and continued to use antibiotics inappropriately. Therefore the campaign was viewed as an important method of increasing awareness of AMR issues.

*I support the concept that antibiotics should not be prescribed willy-nilly. I’m opposed to GPs over-prescribing on all sorts of levels and I think the more people are aware of the potential risks of over-prescribing, inappropriate prescribing, the better.* (Adult Male Interview 6)

In addition to a personal interest, several AGs viewed joining the campaign as part of their professional role. A sense of being blamed for AMR motivated the farmer to join the campaign. The primary care prescriber also disagreed with media coverage which blamed GPs for AMR and an Antimicrobial/Infection Prevention and Control Specialists (AIP) was aware of GPs feeling blamed for AMR. The campaign was valued for its inclusivity and that it did not blame any groups for inappropriately using antibiotics.

*Coming from an industry which is often rather unfairly maligned as being one of the reasons why we’ve got a problem with antibiotics I felt it was particularly important for us to speak out.* (Farmer Male Interview 20)

*I feel a bit peeved that [media] headlines might well read that GPs are doing it [overprescribing antibiotics and contributing to AMR] because I don’t think we are. I mean, you know, I think there are a lot of contributing factors but I don’t think GP prescribing is the sole problem* (Primary Care Prescriber Female Interview 22)

*This is a campaign for everybody, it makes everybody feel that it’s not just gonna blame you know, one side blame the other, which I know has happened a little bit in the past.* (Vet Female Interview 13)

#### Pledge group and pledge choices

Several AGs were eligible for more than one pledge group but the website asked them to select one. AGs chose the pledge group, and their pledge, based on its relevance and likely impact. For some this meant choosing to pledge as part of a wider social or professional group (e.g. dentist, families etc.) rather than within the ‘adult’ pledge group as this was expected to possess greater influence on the awareness and behaviour of others. For example, promoting the campaign to others through their professional or social networks and encouraging appropriate antibiotic use.

*I thought, well, there’s no point in signing up just for myself because it’s nothing that I wouldn’t have done anyway. To pledge for my family means that I’m taking into consideration my son and sort of playing into the bigger picture rather than just like, ‘Oh, my God, my son is ill, therefore the doctor should give him antibiotics’.* (Families Female Interview 5)

*I felt like as a medical student I had more of an advocacy than the others to promote this campaign cos I was meeting people who were more likely to be needing antibiotics cos I’m in an environment where antibiotics are used, so I felt like it was more pertinent.* (Medical student Male Interview 19)

Some AGs described choosing a pledge which required a change to their behaviour, while others commented that their pledge aligned with their current behaviour. However, the AIP AG felt that making such a pledge reinforced and added credibility to their actions.

*I do the first [pledge to consider drainage for dental infections before using antibiotics] anyway, so I could do more good by telling other people about it [pledge to encourage clients/patients and colleagues to become Antibiotic Guardians], so that was the pledge I chose.* (Dentist Female Interview 12)

*I think probably I was doing it before but it kind of, you know, in a way because it’s become a much more public thing it [the campaign] almost gives me the right to challenge and encourage people to do this, so, you know, this is brilliant because I can sort of hang it on there and just say look, you know, this, this is really important and, you know, if you go to this website then you can think about what sort of pledge you’d like to make, I can use that (…) as one way of, you know, somebody doing something, you know, physically doing something to support it.* (AIP Female Interview 17)

The majority of pledges were perceived by AGs to be clear, appropriate, comprehensive and relevant. For example, the pledge recommending treating symptoms for 5 days rather than visiting a GP was generally approved of in relation to the length of time.

*It’s not been five days yet, so I’m gonna wait five days and then I’m gonna go.’ So I think it’s been helpful for that aspect as well, that it’s given me something to work with.* (Adult Female Interview 11)

However, there were mixed responses to the pledge: “For infections that our bodies are good at fighting off on their own, like coughs colds sore throats and flu, I pledge to talk to my pharmacist about how to treat the symptoms first rather than going to the GP”. While some AGs described fulfilling this pledge and perceiving benefits to this approach, others felt that they did not require advice on treating symptoms of minor illnesses but would visit a GP for more severe symptoms because they are able to test for infections and prescribe medication.

*[If] I feel I have a chest infection, which is kind of going above and beyond, the pharmacist is not gonna be able to help me with that. I’m gonna have to go to the GP. I was thinking, I’m unlikely to do that one because I would either not go or I’d need to go to the GP.* (Adult Female Interview 11)

In relation to the pledge about safely disposing of leftover antibiotics, some AGs (adults and pharmacist) questioned why there would be leftover antibiotics as the full course should be taken. Although there was also some recognition that people either save leftover antibiotics for a future infection (Farmer Male Interview 20), already take leftover antibiotics to the pharmacist (Adult Female Interview 11) or have leftover antibiotics when a healthcare professional advises the patient to take prescribed antibiotics only if needed (Adult Female Interview 13).

*Usually antibiotics are prescribed as a full course, so the part about returning any unused antibiotics, I would want them to return it to a pharmacy but I’d want to find out why it was not used in the first place.* (Pharmacist Female Interview 15)

### Pledge recall

Despite an automated email and certificate being sent to all AGs thanking them for participating in the campaign and listing their pledge, the majority could not recall which pledge they had selected and only five could either roughly or fully remember their pledge. For those who could not remember, the interviewer (JK) informed the AG of their pledge before continuing to discuss their choices and the perceived impact of the campaign.

*I know exactly what the campaign is for but I’m struggling to say specifically which pledge I signed up for.* (Medical student Male Interview 19)

### Impact of campaign

The campaign’s impact was discussed at the individual and broader society (UK wide) level and in relation to its visibility and reach.

#### Fulfilling the pledge

The majority of AGs felt they had fulfilled their pledge. The extent to which pledges could be fulfilled was attributed to a number of factors including: pledges matching pre-existing behaviour; not having an opportunity to fulfil the pledge (e.g. not having relevant symptoms since joining the campaign), and; consciously acting in line with pledges (e.g. promoting the campaign to others). Acting in line with the pledge was described by the farmer as an ongoing endeavour. A ‘families’ AG who pledged to encourage their children to sing the ABC song while washing their hands felt the pledge had “really worked” and had become part of their hand washing routine. One dentist and the primary care prescriber perceived the campaign to have contributed to auditing prescribing practice and adherence to prescribing guidelines. The primary care prescriber also perceived the campaign as contributing to on-going changes in the way they prescribe and to a reduced use of antibiotics.

*I haven’t been unwell, you know, in the last twelve months where I’ve thought ‘oh I need to, you know, I need to go and see the pharmacist.’* (Adult Female Interview 8)

*I’ve probably continued down a pathway and a trend that I’d already started (...) with reducing the number of [prescribed] antibiotics.* (Primary Care Prescriber Female Interview 22)

#### Raising and reinforcing awareness

There were mixed reports of the campaign informing AGs about AMR. Some AGs described learning new information including the scale of the AMR issue while others had learnt little and felt the campaign was a continuation of their current thinking.

*I think it’s confirmed what I already know which is always useful because sometimes you can think you know something and you’ve kind of come adrift a bit or of course things change (…) It’s confirmed that what I do is actually the right course of action.* (Pet owner Female Interview 21)

The campaign had prompted AGs to reflect on their personal as well as others’ antibiotic use, and the issue of AMR in general, and maintained their interest in the topic. The campaign reinforced pre-existing beliefs around not taking antibiotics, including refusing them when offered, and led to AGs being more vigilant of others practice. The campaign was also seen as a useful support for professional activities such as teaching medical and dentistry students.

*It’s something I’ve believed for a long time becoming a guardian just emphasised that fact - when there is a suggestion of needing an antibiotic it’s probably made me stronger about not taking the antibiotic.* (Adult Female Interview 7)

#### Promoting the campaign to others

Several AGs had promoted the campaign and its messages to colleagues, students, patients, members of the public, friends and family through informal conversations, lectures, emails, social media and by displaying promotional materials (e.g. posters, certificates and campaign statistics). The campaign was experienced by a few AGs as empowering them to encourage and discuss the issues of the campaign with others. A couple of AGs added the campaign logo and slogan to their email signature, and an AIP AG’s organisation had designed a computer screen saver promoting the campaign and the EAAD.

*I did forward it to people at work and said, ‘I’ve just done this. Are you guys gonna sign up?’ So I was trying to sort of do it that way, via e-mail, for people, so I thought that was a good way to get other people involved in it as well.* (Adult Female Interview 11)

*It’s [campaign certificate displayed on wall of consulting room] a useful reminder for patients, so when I start the ‘look this is a viral infection and it’s not gonna be helped by antibiotics’ I can point to it.* (Primary Care Prescriber Female Interview 22)

*It’s more of a sort of a mental feeling that I’m probably more likely to be involved in discussions around antibiotic resistance, because if someone says, “Oh, what are you doing about it?”, I can say, “Well, actually, I’m signed up, you know - Antibiotic Guardian.” So I have mentioned it to people, that I’m signed up to do it.* (Adult Female Interview 11)

#### Collective action

The campaign was also described as encouraging collective action ensuring that everyone is “singing from the same hymn sheet” and playing their part.

In contrast, two AGs commented that the campaign did not feel like a collective response because it was too selective in how it was promoted.

*I was hoping I was going to become part of a really, really proactive campaign that would get out to the general public more, rather than it seems to be healthcare professionals and people like that that mainly sign up to it and are mainly aware of it. I don’t see too many people, the general public sort of signing up for it, as I say, I talk to a lot of people and they’re just not even aware of it, which is a shame. I think it’s been too, I dunno, selective in where it’s being promoted.* (Adult Female Interview 9)

#### Limited personal impact of campaign

For AGs who perceived themselves as aware of AMR and appropriate antibiotic use, and as acting in line with the selected pledges, the campaign was perceived to have little personal impact. In fact, several AGs commented that they were not the most appropriate target for the campaign and that the campaign is “preaching to the converted.” In addition, a few AGs perceived the campaign to be limited to a day (EAAD). Many had not returned to the website after signing up. Further, an AIP AG had experienced negative feedback from GP’s who viewed the campaign as patronising - “teaching granny to suck eggs.”

*I’m not sure I have used it, per se. As I say, I was already very much anti-antibiotic prescribing on a willy-nilly basis, and being an Antibiotic Guardian (…) or putting my name to the campaign was (1) enforcing the campaign, if you like, by swelling its numbers, (2) giving me a medium which I can refer people to, and (3) I suppose, giving me a sense of gravitas if people would challenge what I was saying.* (Adult Male Interview 6)

*I’m probably already sitting at the end of your bell curve for desirable antibiotic behaviour, so maybe I’m not the right person to be signing these pledges.* (Adult Female Interview 3)

#### Unintended consequences

The medical student commented that the campaign should be careful not to discourage people from seeing the GP when they need to. Indeed, one AG described experiencing feelings of guilt for taking antibiotics after signing up.

*You have to be careful with that because some people are more vulnerable and doing kind of like advice to the whole public could, you wouldn’t want to prevent someone seeing a doctor who actually should see them.* (Medical Student Male Interview 19)

#### Wider impact

While the aims of the campaign were viewed as valuable, several AGs were uncertain about its wider impact. However, it was expected to take time for any measurable effects to develop.

*I think the value of the campaign’s aims are immense (…) for me, apart from, like I said, those few days when I signed up and occasionally when I get reminders about it when I get sent a survey, I couldn’t honestly say that I’d seen the campaign itself have much impact.* (Adult Female Interview 3)

The limited number of pledges displayed on the Campaign’s website, compared with the population as a whole, was interpreted as an estimate of its impact by AGs.

Researcher: What impact do you feel the campaign is having in the UK?

*Participant: Negligible, I would have thought, at that number [of pledges].* (Adult Male Interview 6)

It was suggested that the campaign might reduce antibiotic use and AMR and reduce the workload of GP’s by encouraging patients to visit a pharmacist instead.

*Well if it makes people think ‘do I need to go to a doctor?’ So right away you’re saving on the doctor’s time. Appointments are being freed for people who need them. You’re also saving on the fact that well they haven’t needed the antibiotics, or they went to the pharmacist who is very well qualified to deal with many ailments.* (Adult Female Interview 4)

#### Visibility of the campaign

The campaign and supporting materials were viewed as having poor visibility and the majority of participants had not seen the campaign publicised since joining. The primary care provider was the only AG who described receiving regular email communication about the campaign.

*The campaign was quite – ‘contained’ is the word I’m going to use - and you would think that for something so important they should’ve gone all bells whistling in the media and everything, you know, but it isn’t.* (Pharmacist Female Interview 15)

Several strategies were suggested to increase the reach and publicity of the campaign including: social media promotion; leaflets and posters displayed in doctors’ surgeries, veterinary practices and hospital A&E departments, and; word of mouth communication from HCPs, both about the campaign and prudent use of antibiotics. These measures were suggested to help inform members of the public. An additional strategy suggested was to develop a system for nominating others to join the campaign.

*It’d be good if you could nominate people [laughs], like the Ice Bucket Challenge - have it going through like that - ‘cause there’s definitely a couple of people that could do with taking this pledge, that I’m aware of. So it’s really hard, ‘cause I suppose it doesn’t do the campaign much good if the people that you’re targeting are the people that would have had good behaviours anyway. I suppose who you’re trying to get is people that perhaps could do with a bit of help as to know, ‘Oh yeah, okay, actually, that makes sense. That’s something I should do.’* (Adult Female Interview 11)

#### Follow up communication

Follow-up emails detailing the progress of the campaign and asking the AGs for feedback on their own actions were requested. Follow up emails could emphasise that the website is a resource as well as a place to make pledges.

*You could maybe send out an e-mail in February to say [in sing-song voice], “The coughs and colds season’s nearly over – how did you do with your pledge?” Or, “Did you go to the doctor? Did you go to your pharmacy?” And then maybe that’s where you offer the feedback thing, actually. You could then maybe say you know, “Let us know if you struggled with your pledge,” or “Let us know how you met your pledge and how you found it easy,” or … not easy, but what helps you keep to it, type thing. ‘Cause it could be that you’d get a bunch of e-mails saying, “Yeah, well, I didn’t get a cold, so it was easy.”* (Adult Female Interview 3)

## Discussion

This qualitative evaluation found that the Antibiotic Guardian Campaign offered a framework to support AGs pre-existing beliefs and provided an opportunity for constructive collective action. This is in line with other literature which suggests public campaigns have some positive effects [6] and represent one component of multi-level actions against AMR related issues. This evaluation makes a useful contribution to the limited evidence base on pledge-based interventions [20].

AGs recalled becoming aware of the campaign through professional networks and social media. AGs appeared engaged and concerned with the AMR issue and were motivated to have an impact on AMR. However, despite receiving an automated email with the pledge stated, most AGs could not recall their pledge. Once prompted, many felt they had fulfilled their pledges although this could reflect changing behaviour in line with the pledge, or the pledge reflecting pre-existing behaviour. Others suggested they had not had an opportunity to perform the pledge (for example, by not being ill). The campaign had also led to reflections on AMR related behaviour and diffusion of its messages to others. Although the campaign’s aim was viewed as valuable and promising, its personal and wider impact was unclear to AGs and thought to be limited by restricted visibility and reach. In practice, this restricted visibility can be explained by the absence of sufficient funding to promote the campaign beyond social media and press releases.

Similarities and differences in the findings from the quantitative [10] and qualitative components of the evaluation, and the PHE-led process evaluation, are worthy of note. Unlike the quantitative evaluation, which found that the majority of AGs could partially or fully remember their pledge, only a minority of AGs could recall their pledge during the interview. This may reflect the greater length of time since signing up to the qualitative compared to the quantitative evaluation (10–16 months vs 5 months after the campaign launched), or the different questions asked. The interviews asked AGs to report their exact pledge, whilst the questionnaire asked if they could remember it. The qualitative evaluation suggests the campaign had a more subtle and nuanced impact on behaviour than the quantitative evaluation which described self-reported behaviour change in line with the pledge. The qualitative evaluation suggests the campaign may have supported ongoing changes in behaviour such as prescribing practices, contributed to small changes to health-seeking (e.g. waiting for 5 days before going to the doctor), or reinforced behaviour already aligning with the pledge.

In line with the qualitative findings, the quantitative evaluation also demonstrated that the majority of AGs joined the campaign because of pre-existing awareness of the importance of AMR. The qualitative work supports the quantitative finding that a lack of opportunity to fulfil the pledge was an important reason for not fulfilling it. This relates to the current study’s finding that not developing pledge related symptoms (coughs, colds, sore throats and flu) meant AG’s could not enact the pledge. Both evaluations found mixed reports of acquiring new knowledge through the campaign. The qualitative evaluation builds on the quantitative finding that AGs were willing to promote the campaign to friends, family and colleagues by offering examples of how the campaign has been promoted (e.g. displaying campaign materials). The qualitative findings echo the process evaluation regarding the channels through which AGs become aware of the campaign: professional networks and social media [11].

The campaign’s emphasis on collective action was a motivator for signing up. The importance of collective action to make “resistance visible as a societal threat” has been recommended previously [4]. AGs also valued the emphasis on collective action as opposed to blaming certain individuals or groups for AMR. Attributing blame for AMR among different professional groups, including GPs, farmers and vets [23] as highlighted in this research, is influenced by the unclear evidence on the key drivers of AMR [4]. Brooks and colleagues found that the majority of patients they interviewed blamed antibiotic resistance on other patients and GPs (in the past) for misusing, overusing and prescribing antibiotics [23].

This evaluation captured several accounts of activities to promote the campaign and its messages. The campaign was experienced by a few AGs as empowering them to encourage and discuss the campaign issues with others. This finding could relate to the ‘diffusion of innovation’ theory in which new actions are spread among members of a social system through communication with influential ‘early adopters’ [24]. It is not known whether AGs are influential within their social groups, and it was not within the scope of this evaluation to explore the impact of such diffusion. However, spreading the campaign’s messages by recruiting popular and influential AGs may provide a mechanism for engaging those with less knowledge about AMR. One suggestion from this evaluation is to develop a nomination system for AGs to encourage others to sign up to the campaign.

Other English Antibiotic Campaigns have found low recollection of campaign materials [25]. AGs in this evaluation struggled to recall their pledge but could recall the campaign’s message. A reminder system could be developed to improve recollection of pledge messages. This would also encourage AGs to return to the website and engage with campaign resources. Pledge reminders increased the effectiveness of an intervention targeting healthy eating among school-age children [19]. Greater visibility of the campaign is also likely to be important in facilitating engagement and adherence with pledges [12, 25, 26] and expanding the reach of the campaign beyond those with a personal or professional interest in AMR. However, enhanced visibility is likely to require additional financial support.

An explanation for the limited perceived personal impact of the campaign is the pre-existing AMR concern among AGs. Therefore, the campaign may not have reached those with an opportunity to modify their behaviour. Furthermore, the pledges may not have reflected a goal to which AGs were previously committed but were not achieving, which is fundamental to the ‘intention-behaviour’ gap theory underpinning the campaign. To address this issue, the campaign could develop a system for helping AGs identify an individually tailored, currently unachieved goal to create an appropriate ‘if-then’ scenario [20].

### Strengths and limitations

A strength of this evaluation is the inclusion of a diverse sample reflecting several AG pledge groups and pledges. This allowed the elucidation of campaign experiences from multiple perspectives. However, we experienced a low recruitment rate and the diverse number of pledge groups sampled meant it was not possible to achieve theoretical saturation, when no additional new information is attained [12], across all themes. As noted, the recruited sample contained a higher proportion of female AGs and a lower proportion of HCPs than the full cohort [11], therefore it is possible that the views and experiences of this sample do not reflect those of the full cohort. In addition, our sample does not reflect all possible pledge groups and so there is a potential that these unrepresented groups may have experienced the campaign differently, however this is unlikely given the diverse sample achieved and the similarity of responses.

The qualitative methods used in this evaluation complement and facilitate the interpretation of the quantitative evaluation, generating a more comprehensive impression of campaign experiences. The qualitative work was also conducted later than the quantitative evaluation allowing us to explore the longer-term impacts of the campaign.

Our study is inevitably affected by recall bias, therefore conclusions about the findings must be drawn cautiously. As in the quantitative evaluation, acquiescence bias, whereby participants are aware of the desired responses may have influenced responses, however, AGs suggested improvements to the campaign and reports of the campaign having minimal impact suggest this may not have occurred.

## Conclusions

This qualitative study makes an important contribution to understanding the impact of an innovative online pledge-based campaign seeking to improve the appropriate use of antibiotics. Campaign participation led to some diffusion of campaign messages, behavioural reflections, and evidence of the pledges being accomplished although for many participants this appeared to be through the reinforcement of existing behaviour rather than behaviour change. In line with the quantitative evaluation, we recommend that the campaign be developed further to engage those without pre-existing knowledge of AMR by increasing its visibility, capitalising on the diffusion of the campaign’s message through existing Antibiotic Guardians, particularly those with influence and, consider developing an AG nomination system. The campaign could include more awareness raising content, such as the definition of AMR, for those with limited AMR knowledge. A system for reminding AGs of their pledge could also be developed. Finally, the campaign could develop a system for creating individually tailored pledges relating to committed goals which are currently not being achieved.

## Additional files

Additional file 1: Antibiotic Guardian pledge groups and pledges 2014. (DOCX 27 kb)

Additional file 2: Gender, pledge group, pledge and details of those invited in Phase 1 and 2. (DOCX 17 kb)

## Abbreviations

AG: Antibiotic guardian
AIP: Antimicrobial/Infection Prevention and Control Specialists
AMR: Antimicrobial resistance
EAAD: European Antibiotic Awareness Day
GP: General Practitioner
PHE: Public Health England
UK: United Kingdom
