# Supplementary material for: The Antibiotic Guardian campaign: a qualitative evaluation of an online pledge-based system focused on making better use of antibiotics
Source: BMC Public Health. 2017 Jul 11;18:5. doi: 10.1186/s12889-017-4552-9 (PMC5504645; doi:10.1186/s12889-017-4552-9)
Supplement: Supplementary file 1 — Antibiotic Guardian pledge groups and pledges 2014. (DOCX 27 kb) [file 12889_2017_4552_MOESM1_ESM.docx]

| **Additional file 1.**  **Antibiotic Guardian pledge groups and pledges 2014** | |
| --- | --- |
| **Pledge group** | **Pledges** |
| Healthcare professionals | |
| AMP/IPC | I will create a written action plan for the implementation of SSTF or TARGET within my organisation |
|  | I will encourage and champion members of my organisation to become Antibiotic Guardians |
|  | I will lead promotional activities for EAAD within my organisation |
| Dentists | I will consider drainage for dental infections before issuing antibiotics |
|  | I will encourage clients/patients and colleagues to become Antibiotic Guardians |
|  | When I see a patient with dental pain, I will discuss methods of controlling symptoms rather than prescribing antibiotics as a first course of action |
| Execs / Management / Gov / Commissioners/ Public Health | I pledge to incorporate Antimicrobial Stewardship and Resistance as a quality measure within my commissioning pathways (including Out of Hours) |
|  | I will champion promotional activity for EAAD within my local area or organisation |
|  | I will encourage implementation of Start Smart then Focus guidance within my organisation |
|  | I will ensure that the executive team and board are regularly informed about Antimicrobial Stewardship and AMR in my Trust |
|  | I will visit my local hospital, community pharmacy or general practice surgery to show support for European Antibiotics Awareness Day during EAAD week (18-22 November) |
| Non-Med Prescribers | The next time I want to prescribe an antibiotic for a self-limiting infection for a patient with high expectations for antibiotic treatment, I will offer a delayed/backup prescription first |
|  | When I write an antimicrobial prescription I will make sure it’s in line with local guidelines |
|  | I will undertake one of the recommended CPD activities recommended on the EAAD resources page during EAAD week |
| Nurses | As a nurse prescriber, the next time I decide not to prescribe a patient with an antibiotic for a self-limiting infection, I will give them the TARGET antibiotics patient information leaflet to support their self-care |
|  | As a nurse prescriber, the next time I want to prescribe an antibiotic for a self-limiting infection for a patient with high expectations for antibiotic treatment, I will consider delayed/backup prescription |
|  | The next time I am administering antibiotics, I will prompt the prescriber to review and document a decision 48 hours after the patient has started on antibiotics |
|  | The next time, I am giving a patients antibiotics (on discharge from hospital or via PGD), I will inform the patient on the indication for the antibiotics and that any left-over should be returned to a pharmacy, not shared or reused |
|  | The next time, I see an antibiotic prescription which has continued beyond seven days without specified duration, I will highlight this to the doctors |
| Other HCP | I will champion promotional activity for EAAD within my organisation or local area |
|  | I will encourage clients/patients and colleagues to become Antibiotic Guardians |
|  | In the week of EAAD (18th November) I will take the antibiotic quiz on the EAAD resources |
| Pharmacy Teams | I will check that antibiotic prescriptions comply with local guidance and query those that do not |
|  | The next time a customer presents with a self-limiting infection of coughs/colds I will use the patient information leaflet to explain the potential duration of illness and how to treat their symptoms |
|  | I will undertake one of the recommended CPD activities or e-challenges from the EAAD resources page during EAAD week |
|  | When handing out a prescription that includes antibiotics, I will provide the following key messages/ask the following questions • You have been prescribed antibiotics for xxxx infection (ask if you do not have the information available eg in community pharmacy) • Check if there are any known allergies • Take as prescribed (state dose, frequency and duration) • Do not share your antibiotics with other or reuse them after the stated duration … • Extra self-care information for infection management • Check they have had the flu vaccine (when applicable) |
|  | The next time a customer presents with a self-limiting infection of coughs/colds I will use the patient information leaflet to explain the potential duration of illness and how to treat their symptoms |
| Primary Care Prescribers | When I see a child with a respiratory tract infection (coughs, colds, sore throats, and ear aches) I will use the TARGET - Guide to treat your infection - booklet rather than prescribe antibiotics. Available at http://www.rcgp.org.uk/clinical-and-research/target-antibiotics-toolkit.aspx |
|  | I will ensure all prescribers in my practice including locums have easy access to the local antibiotic guidance |
|  | I will undertake one of the recommended CPD activities on the EAAD resources page during EAAD week |
|  | The next time I intend to prescribe antibiotics for a self-limiting infection to a patient with high expectations of antibiotic treatment, I will use a delayed/backup prescription |
|  | When I see a patient with a self-limiting illness, I will discuss methods of controlling symptoms rather than prescribing antibiotics |
| Secondary Care Prescribers | I will undertake one of the recommended CPD activities on the EAAD resources page during EAAD week |
|  | If I prescribe an antibiotic then I will document indication and duration on the drug chart in line with Start Smart then Focus AMS guidance |
| Veterinary Teams | I will display posters and reading material in my waiting room to help public understanding on antimicrobial resistance |
|  | I will ensure that there are effective cleaning and disinfection protocols in place to minimise the spread of bacteria between patients within the veterinary practice premises |
|  | If a treatment does not appear to work, I will advise a different course of action and report the treatment failure to the Veterinary Medicines Directorate (VMD) |
|  | If there is a need to prescribe antibiotics I will use narrow spectrum drugs wherever possible |
|  | When dispensing antibiotics I will explain the importance of following the labelling instructions and giving the full course |
| **Members of the public** | |
| Adults | It is vital we prevent antibiotics from getting into the environment. I pledge to always take any unused antibiotics to my pharmacy for safe disposal |
|  | For infections that our bodies are good at fighting off on their own, like coughs colds sore throats and flu, I pledge to talk to my pharmacist about how to treat the symptoms first rather than going to the GP |
|  | For infections that our bodies are good at fighting off on their own, like coughs, colds, sore throats and flu, I pledge to try treating the symptoms for five days rather than going to the GP |
| Families | Washing your hands properly is the single best way to prevent the spread of infections. My family pledges to help cut the need for antibiotics by always washing our hands with soap and water for about 30 seconds (about the same time it takes to sing A, B, C, D song) |
|  | For illness that our bodies are good at fighting off on their own, like coughs, colds, sore throats and flu, I pledge to talk to my pharmacist about how to treat my child’s symptoms first rather than going to the GP |
|  | I will visit the ebug website (www.e-bug.eu) with my child(ren) and take one of the antibiotic awareness quizzes together |
| Farmers | To help reduce the need for antibiotics I will evaluate my biosecurity and husbandry measures |
|  | If I have a disease on my farm I will work with my vet to identify preventative measures and help diagnose the cause in order to inform accurate prescribing and stop spread or re-infection |
|  | If my vet prescribes antibiotics I will give them in accordance with the instructions on the label and make sure that the full course is given and that any surplus is returned or disposed of correctly |
| Pet Owners | To help reduce the need for antibiotics I will keep my animal healthy through exercise, good nutrition, relevant vaccination, and by having regular veterinary health checks |
|  | If my pet(s) are prescribed antibiotics by my vet, I will use them as instructed on the label and not give them to another animal |
|  | If my vet prescribes antibiotics I will give them in accordance with the instructions on the label and make sure that the full course is given |
| Students | For infections that our bodies are good at fighting off on their own, like coughs, colds, sore throats and flu, I pledge to talk to my pharmacist about how to treat the symptoms first rather than going to the GP |
|  | For my next clinical case study, I will include one focused on a common infection and how it should be treated in line with local and national guidelines |
|  | I will practice and promote good hand hygiene at all times to reduce transmission of infection and resistance; the WHO FIVE moments for hand-hygiene is recommended http://bit.ly/hand-hygiene-WHO |
|  | The next time I have an opportunity to carry out an audit, I will do one focused on antibiotic use or resistance |
|  | The next time I see an antibiotic prescribed, I will ask the prescriber about the indication and duration, to understand if this is in accordance to local and national guidelines |
|  | The next time I see that a recommended infection prevention practice is not being adhered to (e.g. hand washing), I will respectfully challenge my peers and healthcare workers |

**Antibiotic Guardian pledge groups and pledges 2015**

| **Group** | **Pledges** |
| --- | --- |
| AMP/IPC | I will create a written action plan for the implementation of antimicrobial stewardship guidance (SSTF or TARGET) within my organisation |
|  | I will champion Antibiotic Guardian within my organisation by encouraging at least 10 others to pledge |
|  | I will lead promotional activities for EAAD within my organisation, e.g. by sharing the Antibiotic Guardian quiz, encouraging colleagues to make a pledge and share with their friends and family |
|  | If I see a prescription made outside of local antimicrobial stewardship guidelines I will challenge it and share the local guidance with the prescriber. |
| Dentists | I will consider drainage for dental infections before issuing antibiotics |
|  | I will discuss with patients/clients the importance of antimicrobial resistance and encourage them to take the Antibiotic Guardian quiz and make a pledge to become Antibiotic Guardians |
|  | When I see a patient with dental pain, I will discuss methods of controlling symptoms rather than prescribing antibiotics as a first course of action |
| Executive/ Management/ Government/ Commissioners/ Public Health | Between September and March 2016, I will champion active promotional activity for Antibiotic Guardian within my local area or organisation e.g. set up a laptop/tablet for individuals to pledge |
|  | I will visit my local hospital, community pharmacy, general practice surgery or local vet practices to show support for World Antibiotics Awareness Week (16-22 November) |
|  | I pledge to use local CQUINs to improve antimicrobial stewardship activity within provider organisations |
|  | I pledge to establish an antimicrobial stewardship programme in line with NICE guidance NG15 |
|  | I will ensure that the executive team and board are informed about Antimicrobial Stewardship and AMR in my Trust/Health Board through regular reporting and feedback |
|  | I will familiarise myself with PROTECT, the British Veterinary Society’s 7-point plan or similar guidance materials and resources regarding prudent use of antimicrobials within the animal sector and encourage uptake |
|  | During the cold and flu season, I will add the Antibiotic Guardian electronic signature to all my emails |
|  | I will liaise with my stakeholders and promote prudent use of antibiotics in their sector |
| Nurses | Every time I give antibiotics, I will prompt the prescriber document their reviewing decision after 48 hours |
|  | Every time, I am giving a patients antibiotics, I will inform the patient on the indication for the antibiotics and to take them exactly as prescribed |
|  | Every time, I see an antibiotic prescription which has continued beyond seven days without specified duration, I will highlight this to the prescriber or pharmacist |
|  | I pledge to read and follow the Start Smart then Focus guidance available at: https://www.gov.uk/government/publications/antimicrobial-stewardship-start-smart-then-focus |
| Other HCP | I will encourage clients/patients and colleagues to take the online Antibiotic Guardian quiz and choose their own pledge to become Antibiotic Guardians |
|  | When I write an antimicrobial prescription I will make sure it’s in line with local guidelines |
|  | Every time I want to prescribe an antibiotic for a self-limiting infection for a patient with high expectations for antibiotic treatment, I will offer a delayed/backup prescription first |
|  | I will always record the indication for antibiotic treatment in the patient notes |
|  | I pledge to assess my prescribing practice against the prescribing competences to identify any development needs. |
| Pharmacy Teams | I will check that antibiotic prescriptions comply with local guidance and query those that do not |
|  | Every time a customer presents with a self-limiting respiratory infection I will use the patient information leaflet to explain the potential duration of illness and how to treat their symptoms available at: http://bit.ly/TARGET_patient_leaflets |
|  | When handing out a prescription that includes antibiotics, I will inform the patients of dose and duration and to take their antibiotics exactly as prescribed and to return any unused antibiotics to a pharmacy for safe disposal |
|  | When customers pick up an antibiotic prescription, I will encourage them to take the Antibiotic Guardian quiz online and to choose their own pledge to become Antibiotic Guardians |
| Primary Care Prescribers | When I see a child with a respiratory tract infection I will share the “When should I worry” booklet with parents/carers. Available at http://www.whenshouldiworry.com/ |
|  | I will ensure all prescribers in my practice including locums have easy access to the local antibiotic guidance |
|  | I will adopt the use of delayed/backup prescription for self-limiting respiratory tract infections  The next time I intend to prescribe antibiotics for a self-limiting infection to a patient with high expectations of antibiotic treatment, I will use a delayed/backup prescription |
|  | When I see a patient with a self-limiting respiratory tract infection, I will discuss methods of self-care and safety netting rather than prescribing antibiotics and share the TARGET “Treating your infection” leaflet available at http://www.rcgp.org.uk/clinical-and-research/toolkits/target-antibiotics-toolkit.aspx |
|  | I will review my practice prescribing against that of the CCG and national averages on fingertips. |
| Secondary Care Prescribers | I will undertake one of the recommended CPD activities on the EAAD resources page during EAAD week |
|  | If I prescribe an antibiotic then I will document indication, duration and review dates on the drug chart in line with Start Smart then Focus AMS guidance |
|  | I will review and share my Trusts prescribing data with my colleagues and use this to inform my own prescribing |
| Veterinary & Nurses Teams | I will ensure clients have and understand all the information they need to administer the antibiotic correctly to their animal(s) per the prescription or instruction as well as understand the importance of following the instructions on the label |
|  | I will ensure that there are effective and up to date cleaning and disinfection protocols in place to minimise the spread of bacteria between patients within the veterinary practice premises |
|  | I will promote culture and sensitivity testing before prescribing antibiotics wherever possible and I will report any suspected antibiotics treatment failure to the Marketing Authorisation Holder (MAH) or the Veterinary Medicines Directorate (VMD) |
|  | If there is a need to prescribe antibiotics I will use narrow spectrum drugs wherever possible |
|  | I will reduce the reliance on prophylactic use of antibiotics in farm animals by working with farm clients to produce a clear animal health plan which encourages good bio-security and husbandry practices |
|  | I will maintain awareness of antimicrobial resistance through continuous professional development (CPD) and promote a culture of responsible use of antibiotics at work and within my professional network |
| Midwives | When a mother is prescribed antibiotic, I will ensure that she understands why they have been prescribed and to how take them |
|  | If a mother presents with a self-limiting illness such as a cold, I will ensure she understand that antibiotics are unnecessary and has information on how to treat her symptoms and when to seek further advice |
|  | When I write an antibiotic prescription, I will make sure that it is in line with local guidelines |
| **Members of the Public** | |
| Adults | It is vital we prevent antibiotics from getting into the environment. I pledge to always take any unused antibiotics to my pharmacy for safe disposal |
|  | For infections that our bodies are good at fighting off on their own, like coughs colds sore throats and flu, I pledge to talk to my pharmacist about how to treat the symptoms first rather than going to the GP |
|  | For infections that our bodies are good at fighting off on their own, like coughs, colds, sore throats and flu, I pledge to try treating the symptoms for five days rather than going to the GP |
|  | If the NHS offers me a flu vaccination, I pledge to accept. |
|  | If I’m prescribed antibiotics, I will take them exactly as prescribed and never share them with others |
| Families | Sing the ABC song when washing our hands with soap and water. Washing hands properly (at least 30 seconds), especially before eating, is the single best way to prevent the spread of infections and keep your family healthy |
|  | When I think my child has a likely respiratory infection, I pledge to talk to my pharmacist about how to treat my child’s symptoms first rather than going to the GP |
|  | I will visit the ebug website (www.e-Bug.eu) with my child(ren) and take one of the antibiotic awareness quizzes together |
|  | If anyone in my family is prescribed antibiotics, I will ensure they are taken exactly as prescribed and never shared with others |
|  | If the NHS offers a flu vaccination for individuals in my family, we pledge to accept. |
| Farmers | To help reduce the need for antibiotics I will work with my veterinary surgeon to keep an up to date Animal Health Plan. This will include, if necessary, changes to farm practices such as biosecurity. |
|  | If I have a disease on my farm, I will work with my vet to identify the cause and any preventative measures which could stop spread or re-infection, so as to reduce the need for antibiotics in the future. |
|  | If my vet prescribes antibiotics, I will give them in accordance with the instructions provided – e.g. dose, withdrawal period – and make sure that unused or expired products are returned or disposed of correctly and not given to animal(s) for which they have not been prescribed. |
|  | I will keep full record of all medicines purchased and used in my animals – including in-feed and in-water medicines. |
|  | I will annually review and discuss the antibiotic use on my farm with my vet, and look for ways of optimising my use as necessary. |
|  | I will keep my animal(s) healthy through good nutrition and husbandry, relevant vaccination and worming and by having regular veterinary health checks. |
| Pet and Horse Owners | To help reduce the risk of my animal(s) getting an infection which requires antibiotic treatment, I will keep them healthy through exercise, good nutrition, relevant vaccination, suitable accommodation and by having regular veterinary health checks |
|  | If my vet prescribes antibiotics for my animal(s), I will follow the instructions provided and I will not change the dosage or stop the therapy early. |
|  | If my vet prescribes antibiotics to my animal(s), I will not re-use antibiotics prescribed for an earlier illness or give them to other animals or animal owners and I will dispose of unused or expired products appropriately. |
|  | I will not request antibiotics if my vet has not recommended or prescribed them. |
|  | I will support vets’ efforts to reduce the risk of antimicrobial resistance by better understanding where and when antibiotics are needed e.g. by being familiar with the Protect ME campaign from BEVA. |
| UK and International Organisations | Between September and November 2015, we will champion/plan key promotional activities for EAAD and first World Antibiotic Awareness Week within our local area and to members of our organisation |
|  | As an organisation, we will work to contribute or help develop an action plan for our membership or a national action plan for our country on antimicrobial resistance in line with the global plan |
|  | In support of the first WAAW, our organisation will share another country’s AMR messages to demonstrate global efforts to reduce AMR. |
|  | As a public health institution, when we release materials on AMR, we will aim to use language which will minimise public misconceptions on drug-resistant infections |
| **Students and Educators** | |
| Students | The next time I see an antibiotic prescribed, I will ask the prescriber about the indication and duration, to understand if this is in accordance to local and national guidelines |
|  | The next time I see that a recommended infection prevention practice is not being adhered to (e.g. hand hygiene, on-farm biosecurity), I will respectfully challenge my peers and healthcare workers/veterinarians |
|  | I will use the e-Bug young adult peer education materials on drug-resistant infections, antibiotic use and vaccinations to educate and inform my peers. Available at: <http://bit.ly/ebug_peer-edu> |
|  | I will familiarise myself with PROTECT, the British Veterinary Society’s 7-point plan or similar guidance materials and resources regarding prudent use of antimicrobials within the animal sector and discuss them in my classes |
|  | I will ask my tutors to discuss in class the challenges of responsible use of antibiotics in various animal sectors, including companion animals |
|  | If I’m prescribed antibiotics, I will take them exactly as prescribed and never share them with others |
|  | For infections that my body can fight off on its own, like coughs, colds, sore throats and flu, I pledge to talk to my pharmacist about how to treat my symptoms first rather than going to the GP |
|  | I will wash my hands after sneezing or coughing to reduce transmission of infection and antibiotic resistance |
| Educators | When I’m teaching my pupils about science, I will visit the e-Bug website (www.e-Bug.eu) for resources to help develop a lesson plan for my pupils to educate them on antimicrobial resistance |
|  | As part of my next health-related lecture, I will get my students to complete the Antibiotic Guardian online quiz and encourage them to choose their own pledge and become Antibiotic Guardians. |
|  | I will encourage my students to impart their knowledge, challenge their peers to take the Antibiotic Guardian quiz online and choose a pledge to become an Antibiotic Guardian. |
|  | I will visit a local school or college and use the full e-Bug website peer education antibiotic materials to teach junior or senior students about antibiotics and resistance. Available at: http://bit.ly/ebug_peer-edu |
