# Supplementary material for: The Antibiotic Guardian campaign: a qualitative evaluation of an online pledge-based system focused on making better use of antibiotics
Source: BMC Public Health. 2017 Jul 11;18:5. doi: 10.1186/s12889-017-4552-9 (PMC5504645; doi:10.1186/s12889-017-4552-9)
Supplement: Supplementary file 2 — Gender, pledge group, pledge and details of those invited in Phase 1 and 2. (DOCX 17 kb) [file 12889_2017_4552_MOESM2_ESM.docx]

| **Additional file 2.**  **Gender, pledge group, pledge and details of those invited in Phase 1 and 2** |  |
| --- | --- |
| **Phase 1 invited sample** | **n** |
| **Gender** |  |
| *Male* | 7 |
| *Female* | 41 |
|  |  |
| **Pledge group** |  |
| Healthcare professional or leader  *Antimicrobial/Infection Prevention and Control Specialists* | 6 |
| *Dentists* | 3 |
| *Nurses* | 1 |
| *Other Healthcare Workers (eg Podiatrists, chiropodists, radiographers, therapists, social workers)* | 3 |
| *Pharmacy Teams* | 10 |
| *Primary Care Prescribers* | 2 |
| *Secondary Care Prescribers* | 2 |
| *Veterinary Practitioners* | 3 |
| *Student (medical)* | 1 |
|  |  |
| Members of the public |  |
| *Adults* | 16 |
| *Families* | 2 |
|  |  |
| **Pledge** |  |
| As a nurse prescriber, the next time I decide not to prescribe a patient with an antibiotic for a self-limiting infection, I will give them the TARGET antibiotics patient information leaflet to support their self-care | 1 |
| For illness that our bodies are good at fighting off on their own, like coughs, colds, sore throats and flu, I pledge to talk to my pharmacist about how to treat my child’s symptoms first rather than going to the GP | 1 |
| For infections that our bodies are good at fighting off on their own, like coughs colds sore throats and flu, I pledge to talk to my pharmacist about how to treat the symptoms first rather than going to the GP | 10 |
| I will champion promotional activity for EAAD within my organisation or local area | 2 |
| I will check that antibiotic prescriptions comply with local guidance and query those that do not | 4 |
| I will consider drainage for dental infections before issuing antibiotics | 1 |
| I will create a written action plan for the implementation of SSTF or TARGET within my organisation | 2 |
| I will encourage and champion members of my organisation to become Antibiotic Guardians | 4 |
| I will ensure all prescribers in my practice including locums have easy access to the local antibiotic guidance | 1 |
| I will lead promotional activities for EAAD within my organisation | 2 |
| I will undertake one of the recommended CPD activities on the EAAD resources page during EAAD week | 3 |
| If a treatment does not appear to work, I will advise a different course of action and report the treatment failure to the Veterinary Medicines Directorate (VMD) | 1 |
| If my pet(s) are prescribed antibiotics by my vet, I will use them as instructed on the label and not give them to another animal | 1 |
| If there is a need to prescribe antibiotics I will use narrow spectrum drugs wherever possible | 2 |
| In the week of EAAD (18th November) I will take the antibiotic quiz on the EAAD resources | 1 |
| It is vital we prevent antibiotics from getting into the environment. I pledge to always take any unused antibiotics to my pharmacy for safe disposal | 4 |
| The next time I see that a recommended infection prevention practice is not being adhered to (e.g. hand washing), I will respectfully challenge my peers and healthcare workers | 1 |
| Washing your hands properly is the single best way to prevent the spread of infections. My family pledges to help cut the need for antibiotics by always washing our hands with soap and water for about 30 seconds (about the same time it takes to sing A, B, C, D song) | 1 |
| When handing out a prescription that includes antibiotics, I will provide the following key messages/ask the following questions • You have been prescribed antibiotics for xxxx infection (ask if you do not have the information available eg in community pharmacy) • Check if there are any known allergies • Take as prescribed (state dose, frequency and duration) • Do not share your antibiotics with other or reuse them after the stated duration … • Extra self-care information for infection management • Check they have had the flu vaccine (when applicable) | 6 |
| **Total** | **48** |

| **Phase 2 invited sample** | **n** |
| --- | --- |
| **Gender** |  |
| Male | 14 |
| Female | 32 |
|  |  |
| **Pledge group** |  |
| Healthcare professional or leader |  |
| *Primary Care Prescribers* | 7 |
| *Secondary Care Prescribers* | 7 |
| *Antimicrobial/Infection Prevention and Control Specialists* |  |
| *Pharmacy Teams* |  |
| *Nurses* | 7 |
| *Dentists* |  |
| *Veterinary Practitioners* |  |
| *Other Healthcare Workers (e.g. Podiatrists, chiropodists, radiographers, therapists, social workers)* | 7 |
| *Students (Medical, Pharmacy, Dental, Veterinary, Other)* | 7 |
|  |  |
| Members of the public |  |
| *Farmers* | 5 |
| *Pet Owners* | 5 |
| *Students (non-medical)* | 1 |
|  |  |
| **Pledge** |  |
| As a nurse prescriber, the next time I decide not to prescribe a patient with an antibiotic for a self-limiting infection, I will give them the TARGET antibiotics patient information leaflet to support their self-care | 1 |
| For infections that our bodies are good at fighting off on their own, like coughs, colds, sore throats and flu, I pledge to talk to my pharmacist about how to treat the symptoms first rather than going to the GP | 4 |
| I will champion promotional activity for EAAD within my organisation or local area | 1 |
| I will check that antibiotic prescriptions comply with local guidance and query those that do not | 1 |
| I will encourage clients/patients and colleagues to become Antibiotic Guardians | 4 |
| I will ensure all prescribers in my practice including locums have easy access to the local antibiotic guidance | 2 |
| I will practice and promote good hand hygiene at all times to reduce transmission of infection and resistance; the WHO FIVE moments for hand-hygiene is recommended http://bit.ly/hand-hygiene-WHO | 3 |
| I will undertake one of the recommended CPD activities on the EAAD resources page during EAAD week | 3 |
| If I prescribe an antibiotic then I will document indication and duration on the drug chart in line with Start Smart then Focus AMS guidance | 5 |
| If my vet prescribes antibiotics I will give them in accordance with the instructions on the label and make sure that the full course is given | 3 |
| In the week of EAAD (18th November) I will take the antibiotic quiz on the EAAD resources | 2 |
| The next time I am administering antibiotics, I will prompt the prescriber to review and document a decision 48 hours after the patient has started on antibiotics | 1 |
| The next time I see an antibiotic prescribed, I will ask the prescriber about the indication and duration, to understand if this is in accordance to local and national guidelines | 1 |
| The next time, I am giving a patients antibiotics (on discharge from hospital or via PGD), I will inform the patient on the indication for the antibiotics and that any left-over should be returned to a pharmacy, not shared or reused | 1 |
| The next time, I see an antibiotic prescription which has continued beyond seven days without specified duration, I will highlight this to the doctors | 4 |
| To help reduce the need for antibiotics I will evaluate my biosecurity and husbandry measures | 2 |
| To help reduce the need for antibiotics I will keep my animal healthy through exercise, good nutrition, relevant vaccination, and by having regular veterinary health checks | 4 |
| When I see a patient with a self-limiting illness, I will discuss methods of controlling symptoms rather than prescribing antibiotics | 4 |
| **Total** | **46** |
